# Recognition and Grasping of Disorderly Stacked Wood Planks Using a Local Image Patch and Point Pair Feature Method

**DOI:** 10.3390/s20216235

**Published:** 2020-10-31

**Authors:** Chengyi Xu, Ying Liu, Fenglong Ding, Zilong Zhuang

**Affiliations:** 1 College of Mechanical and Electronic Engineering, Nanjing Forestry University, Nanjing 210037, China; xucy312@163.com (C.X.); dfl@njfu.edu.cn (F.D.); zzl0702@njfu.edu.cn (Z.Z.); 2 College of Mechanical Engineering, Nantong Vocational University, Nantong 226007, China

**Keywords:** convolutional auto-encoders, local image patch, point pair feature, plank recognition, robotic grasping

## Abstract

Considering the difficult problem of robot recognition and grasping in the scenario of disorderly stacked wooden planks, a recognition and positioning method based on local image features and point pair geometric features is proposed here and we define a local patch point pair feature. First, we used self-developed scanning equipment to collect images of wood boards and a robot to drive a RGB-D camera to collect images of disorderly stacked wooden planks. The image patches cut from these images were input to a convolutional autoencoder to train and obtain a local texture feature descriptor that is robust to changes in perspective. Then, the small image patches around the point pairs of the plank model are extracted, and input into the trained encoder to obtain the feature vector of the image patch, combining the point pair geometric feature information to form a feature description code expressing the characteristics of the plank. After that, the robot drives the RGB-D camera to collect the local image patches of the point pairs in the area to be grasped in the scene of the stacked wooden planks, also obtaining the feature description code of the wooden planks to be grasped. Finally, through the process of point pair feature matching, pose voting and clustering, the pose of the plank to be grasped is determined. The robot grasping experiment here shows that both the recognition rate and grasping success rate of planks are high, reaching 95.3% and 93.8%, respectively. Compared with the traditional point pair feature method (PPF) and other methods, the method present here has obvious advantages and can be applied to stacked wood plank grasping environments.

## 1. Introduction

At present, the grasping process of disorderly stacked wooden planks is mainly completed by manual methods, such as the sorting and handling of wooden planks, and the paving of wooden planks. There are existing defects, such as low labor and high cost. With the rapid development and wide application of robotic technology, vision-based robot intelligent grasping technology has high theoretical significance and practical value to complete this work. The visual recognition and positioning of disorderly stacked wooden planks is an important prerequisite for successful robot grasping. The vision sensor is the key component of the implementation. A RGB-D camera can be regarded as a combination of a color monocular camera and a depth camera, and this camera can collect both texture information and depth information at the same time [1], which has obvious application advantages.

The recognition, detection, and location of objects is a research hotspot in both Chinese and overseas contexts. Hinterstoisser et al. [2] extracted features from the color gradient of the target and the normal vector of the surface and matched them to obtain a robust target detection result. Rios-Cabrera et al. [3] used a cluster to find the target and the detection speed was faster. Rusu et al. [4] calculated the angle between the normal vector of a point cloud and the direction of the viewpoint based on the viewpoint feature histogram (VFH), but this was not robust to occlusion problems. The orthogonal viewpoint feature histogram (OVFH) [5] was proposed. Birdal et al. [6,7,8] used a point cloud to extract point pair features (PPFs), and used shape information to identify and detect targets, but this was mainly for objects with complex shapes. Lowe et al. [9,10] proposed a scale-invariant 2D feature point the scale invariant feature transform method (SIFT) local feature point with good stability, but it was not suitable for multiple similar targets in the same scene. There are also the feature descriptors surf [11], spin image [12], signature of histogram of orientation (SHOT) [13], etc., Choi [14] added color information on the basis of traditional four-dimensional geometric point pair features and obtained better accuracy than the original PPF method. Ye et al. [15] proposed fast hierarchical template Matching Strategy of Texture-Less Objects, which takes less time than the origin method. Muñoz et al. [16] proposed a novel part-based method using an efficient template matching approach where each template independently encodes the similarity function using a forest trained over the templates. In reference to the multi-boundary appearance model, Liu [17] proposed to fit the tangent to the edge point of the model as the direction vector of the point. Through the four-dimensional point pair feature matching and positioning, a good recognition result was obtained. Li [18] proposed a new descriptor curve set feature (CSF), where the descriptor curve set feature describes a point by describing the surface fluctuations around the point and can evaluate the pose. CAD-based pose estimation was also used to solve recognition and grasping problems. Wu et al. [19] proposed constructing 3D CAD models of objects via a virtual camera, which generates a point cloud database for object recognition and pose estimation. Chen et al. [20] proposed a CAD-based multi-view pose estimation algorithm using two depth cameras to capture the 3D scene.

Recently, deep learning has been applied to the recognition and grasping of robot situations. Kehl [21] proposed the use of convolutional neural networks for end-to-end training to obtain the pose of an object. Caldera et al. [22] proposed a novel approach to multi-fingered grasp planning leveraging learned deep neural network models. Kumara et al. [23] proposed a novel robotic grasp detection system that predicts the best grasping pose of a parallel-plate robotic gripper for novel objects using the RGB-D image of the scene. Levine et al. [24] proposed a learning-based approach to hand-eye coordination for robotic grasping from monocular images. Zeng et al. [25] a robotic pick-and-place system that is capable of grasping and recognizing both known and novel objects in cluttered environments. Kehl [26] proposed a 3D object detection method that used regressed descriptors of locally-sampled RGB-D patches for 6D vote casting. Zhang [27] proposed a recognition and positioning method that uses deep learning to combine the overall image and the local image patch. Le et al. [28] proposed applying an instance segmentation-based deep learning approach using 2D image data for classifying and localizing the target object while generating a mask for each instance. Tong [29] proposed a method of target recognition and localization using local edge image patches. Jiang et al. [30] used a deep convolutional neural network (DCNN) model that was trained on 15,000 annotated depth images synthetically generated in a physics simulator to directly predict grasp points without object segmentation.

Even in the current era of deep learning, the point pair feature (PPF) method [7] has a strong vitality in bin-picking problem. Its algorithm performance is still no less than that of deep learning. Many scholars have made a lot of improvements to PPF [6,7,8,14,15,31] because of its advantages. The general framwork of PPF has not changed significantly at the macro or micro level anyway. Vidal et al. [31] proposed an improved matching method for Point Pair Features with the discriminative value of surface information.

At present, most of the robot visual recognition and grasping scenes are put together with different objects and the target objects that need to be recognized have rich contour feature information. However, the current research methods are not effective for the identification and grasping of disorderly stacked wooden planks. The main reason for this is that the shape of the wooden plank itself is regular and symmetrical and is mainly a large plane. The contour change information is not rich and different planks have no obvious or special features. There are many similar features such as shapes and textures. When these wooden planks are stacked together, it is difficult to identify and locate one of the wooden planks using conventional methods, making it difficult for a robot to grasp a plank when among many. Therefore, we utilize PPF combined other features to recognize and locate unordered stacked planks.

**Figure 1:**
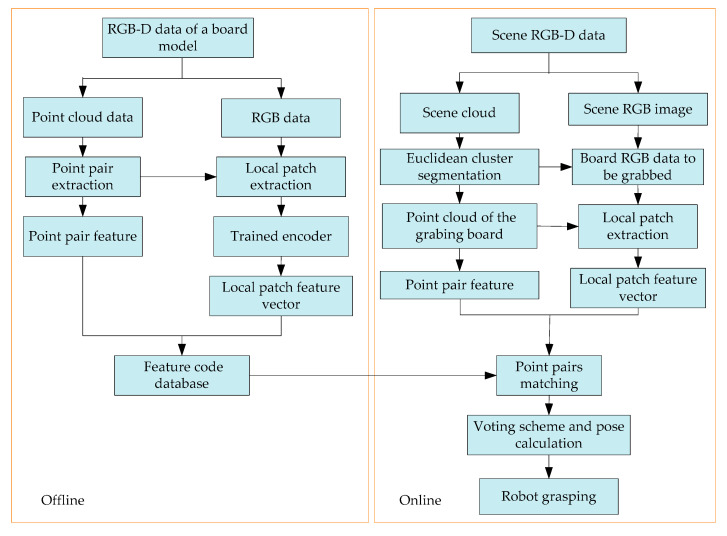
The overall algorithm flow of robot grasping.

The local image patches of the wooden plank images both from the self-developed scanning equipment and the disorderly stacking plank scene here are taken as a data set. Using the strong fitting ability of a deep convolutional autoencoder, the convolutional autoencoder is trained to obtain stable local texture feature descriptors. The overall algorithm flow realized by robot grasping is shown in Figure 1. In the offline stage, a pair of feature points are randomly selected on the wooden plank image and local image patches are intercepted. These two image patches are sequentially input to the trained feature descriptor to obtain the local feature vector and combine the geometric feature information of the point pair to build the feature code database of the plank model. In the online stage, the disorderly stacking plank scene is segmented and the plank area to be grasped is extracted, and the geometric feature information of the point pair is also extracted and the feature description code of the local image patch is found, similar to the offline stage. Then, point pair matching is performed with the established plank feature database. Finally, the robot is used to realize the positioning and grasping operation after all the point pairs complete pose voting and pose clustering.

## 2. Methods of the Local Feature Descriptor Based on the Convolutional Autoencoder

**Figure 2:**
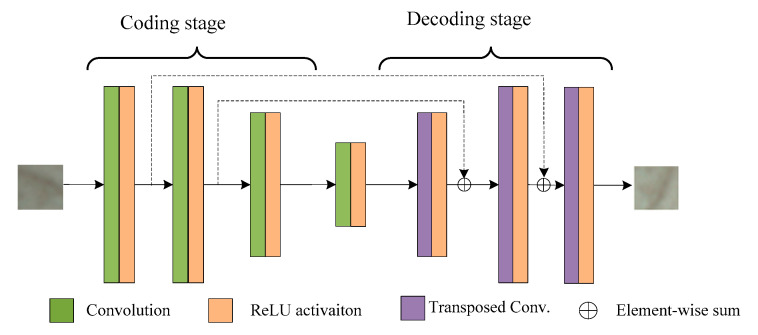
Convolutional auto-encoder mode.

Traditional autoencoders [32,33,34] are generally fully connected, which will generate a large number of redundant parameters. The extracted features are global, local features are ignored, and local features are more important for wood texture recognition. Convolutional neural networks have the characteristics of local connection and weight sharing [35,36,37,38,39,40], which can accelerate the training of the network and facilitate the extraction of local features. The deep convolutional autoencoder designed in this paper is shown in Figure 2. The robot drives the camera to collect small image patches at different angles around the same feature point in the scene of disorderly stacked wood planks as the input and expected value of output to train the convolutional autoencoder. Its network is mainly composed of two stages, namely, encoding and decoding. The encoding stage has four layers. Each layer implements feature extraction on input data convolution and ReLU activation function operations. There are also four layers in the decoding stage. Each layer implements feature data reconstruction through operations such as transposed convolution and ReLU activation function operations. This network model combines the advantages of traditional autoencoders and convolutional neural networks, in which residual learning connections are added to improve the performance of the network.

In the encoding stage, the input of the convolutional layer has D groups of feature matrices, and the two-dimensional M×M feature matrix of the dth group is $x^{d}\;,1\leq d\leq D$. The two-dimensional N×N convolution kernel of the group of the $k{}^{\prime }$th channel of the output convolution layer is $\;W^{k,d}$, and the $k{}^{\prime }$th feature map of the output convolution layer is $h^{k}\in R^{M^{\prime }\times M^{\prime }}$, where $M^{\prime }=M-N+1$, where $h^{k}$ can then be expressed as follows:

(1) $$h^{k}=f\left(\overset{D}{\underset{d=1}{\sum }}x^{d}*W^{k,d}+b^{k}\right)$$

In Equation (1), f is the activation function, * is the two-dimensional convolution, and $b^{k}$ is the offset of the $k{}^{\prime }$th channel of the convolutional layer.

The decoding stage is designed to reconstruct the feature map obtained in the encoding stage, input a total of K groups of feature matrices, and output the dth feature map, $y^{d}\in R^{M^{\prime \prime }\times M^{\prime \prime }}$, where $M^{\prime \prime }=M^{\prime }-N+1$. Below, $y^{d}$ can be expressed as follows:

(2) $$y^{d}=f\left(\overset{K}{\underset{k=1}{\sum }}h^{k}*{\tilde{W}}^{k,d}+c^{d}\right)$$

In Equation (2), ${\tilde{W}}^{k,d}$ is horizontal and vertical flip of the convolution kernel $W^{k,d}\;$ and 1≤k≤K. Additionally, $c^{d}$ is the offset of the $d{}^{\prime }$th channel of the deconvolution layer.

The convolutional autoencoder encodes and decodes the original intercepted image to continuously learn the parameters and offsets of the convolution kernel in the network, so that the output result y is as close as possible to the given output expectation $x^{\prime }$, and to minimize the reconstruction error function in Equation (3)

(3) $$E=\frac{1}{2n}\sum_{i=1}^{n}{\left\|y_{i}-{x^{\prime }}_{i}\right\|}^{2}$$

where n is the number of samples input into the model training, $y_{i}$ represents the actual output samples, and $x^{\prime }$ represents the expected value of the output.

A back propagation (BP) neural network error back propagation algorithm is used to adjust the network parameters. If the training result makes the autoencoder converge, the trained autoencoder encodes the part of the network to obtain a local texture feature descriptor of the wood plank that is robust to changes in perspective, that is, the local image patch is input into the coding part of the convolutional encoder to get the corresponding feature description code.

**Figure 3:**
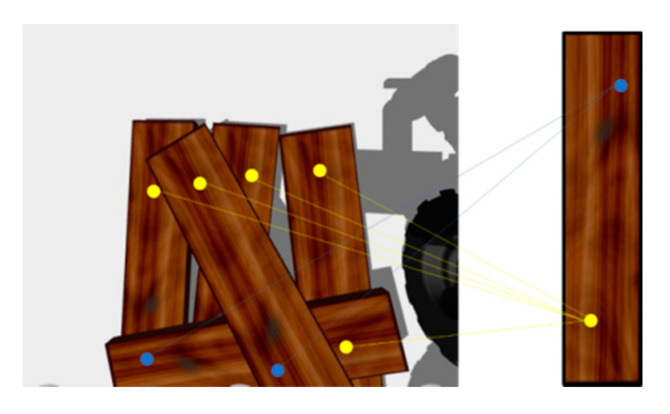
Local image patches matching.

**Figure 4:**
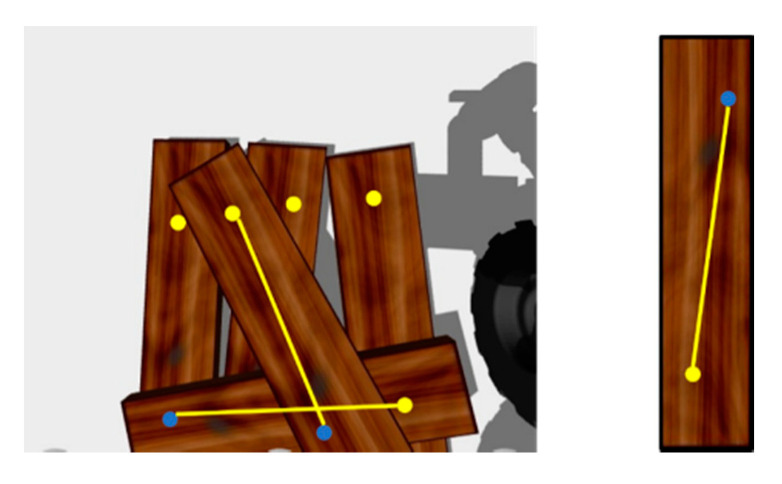
Local image patches of paired relation.

Only the local image features of the wooden planks are used for the matching and recognition of a single plank. Since there are multiple and similar wooden planks in the visual scene of stacked wooden planks, the algorithm cannot identify whether multiple local image patch features belong to the same wooden plank, and they are likely to be scattered on different wooden planks, as shown in Figure 3. This is very easy to mismatch. We hope local image patches on the same board to be pair relations, as shown in Figure 4.

## 3. Offline Calculation Process: Model Feature Description Using Point Pair Features and Local Image Patch Features

**Figure 5:**
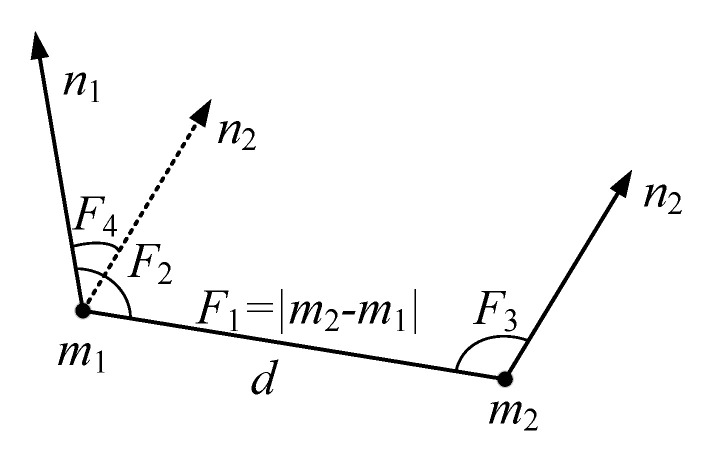
Schematic diagram of point pair features.

Consider adding a point pair geometric feature constraint relationship to two points on the same wooden plank model, as shown in Figure 5.

For any two points $m_{1}$ and $m_{2}$ and their respective normal vectors $,\;n_{1},n_{2}$, define the constraint relationship describing the local texture feature point pair as shown in Equation (4) [7]:

(4) $$F\left(m_{1},m_{2}\right)=\left[\left\|d\right\|,∠\left(n_{1},d\right),∠\left(n_{2},d\right),∠\left(n_{1},n_{2}\right)\right]$$

where ‖d‖ is the distance between the two points, $∠\left(n_{1},d\right)$ is the angle between the normal vector $n_{1}$ and line d connecting two points, $∠\left(n_{2},d\right)$ is the angle between the normal vector $n_{2}$ and the line d connecting two points, and $∠\left(n_{1},n_{2}\right)$ is the angle between the normal vector $n_{1}$ and the normal vector $n_{2}$. Then, namely:

(5) $$\{\begin{array}{c} F_{1}=\left\|d\right\|=\left|m_{2}-m_{1}\right|\\ F_{2}=∠\left(n_{1},d\right)=\arccos\frac{n_{1}\cdot d}{\left|n_{1}\right|\left|d\right|}\\ F_{2}=∠\left(n_{2},d\right)=\arccos\frac{n_{2}\cdot d}{\left|n_{2}\right|\left|d\right|}\\ F_{3}=∠\left(n_{1},n_{2}\right)=\arccos\frac{n_{1}\cdot n_{2}}{\left|n_{1}\right|\left|n_{2}\right|} \end{array}$$

We at first performed point pair feature sampling on the point cloud of a single plank model, that is, we first took a feature point, established a point pair relationship with all other feature points in turn, and then took a new feature point to establish a point pair relationship, repeating the execution until the point pair relationship of all feature points was established, and the characteristic parameter F was calculated for each pair of non-repeated point pairs.

Since the shape of the plank itself is mainly a large rectangular plane, with symmetrical regularity, the corresponding characteristic parameter information of different points on a single plank is the same or similar. Pointpair features alone cannot complete the uniqueness of the plank feature matching, so the feature of the local image patch of the point-pair was added here. We input the intercepted local image patch into the previously trained encoder, where the encoder only needed to take the encoding part of the original convolutional encoder, the feature vector corresponding to the input local image patch can be obtained, and the feature vector of all non-repetitive point pairs of the local image patch can be combined with the point pair feature geometric information. 

**Figure 6:**
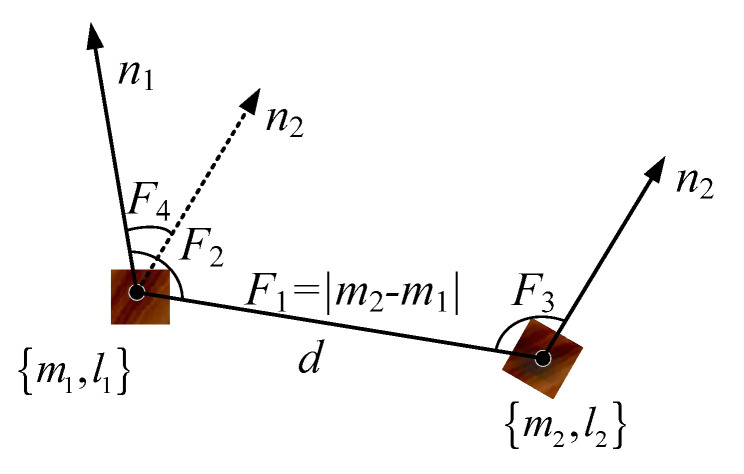
Local patch point pair feature (LPPPF).

As shown in Figure 6, we define a more comprehensive description of the characteristic parameters of the wood plank, namely:

(6) $$F_{l}\left(m_{1},m_{2}\right)=\left[\left\|d\right\|,∠\left(n_{1},d\right),∠\left(n_{2},d\right),∠\left(n_{1},n_{2}\right),l_{1},l_{2}\right]$$

where $l_{1},l_{2}$ represent the feature code extracted from the local image patch by the encoder.

**Figure 7:**
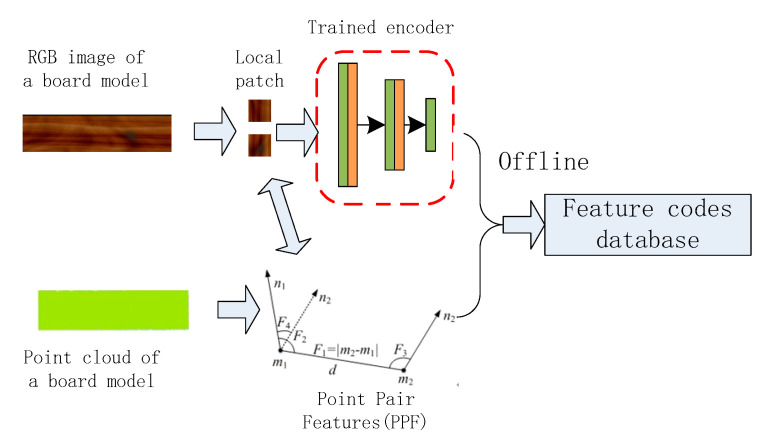
Establishment of feature code database of the wood plank model.

The KD-tree (k-dimensional tree) method is used to build a feature code database reflecting the characteristics of the wood plank model, as shown in Figure 7. In summary, the model description method that combines point pair features and image local features can avoid the drawbacks of using one of the methods alone.

## 4. Online Calculation Process

### 4.1. Generating the Feature Code of the Plank to Be Grasped

**Figure 8:**
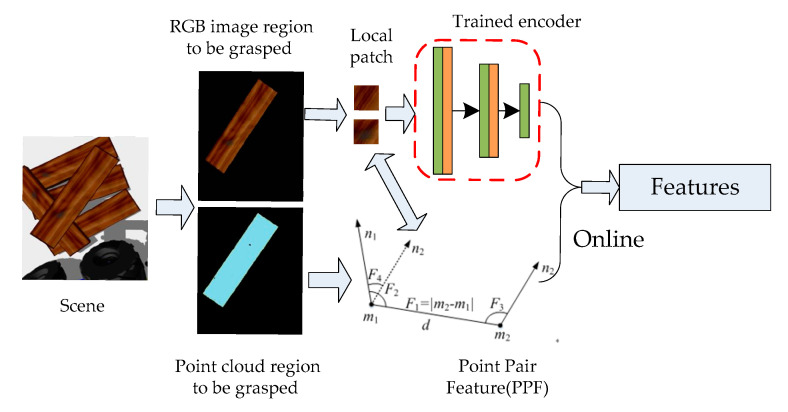
Generation of feature code of the wood plank to be grasped.

Since the stacked wooden planks have obvious hierarchical characteristics, the robot’s grasping process is generally carried out in order from top to bottom, and the wooden plank grasped each time should be the top layer in the scene at that time. First, the Euclidean distance cluster method [41] was used to segment the scene under robot vision, then calculating the average depth of different clusters and selecting the smallest average depth value as the area to be grasped. As shown in Figure 8, there is no need to match all the wood planks in the scene later, which saves on the computing time. Next, we randomly extracted a part of the scene pointpair feature information and the point pair corresponding local image patches from the area to be grasped, and input the local image patches into the trained encoder to generate local image patch feature vectors. These were combined with the pointpair geometric feature information to form a feature description code.

### 4.2. Plank Pose Voting and Pose Clustering

We extracted point pairs that were similar to the feature codes generated in the scene from the feature code database established offline and measured their similarity by the Euclidean distance to complete the point pair matching:

(7) $$dist(F_{off},F_{i})={\left\|F_{off}-F_{i}\right\|}_{2}$$

where $F_{i}$ represents the feature code extracted and generated in the scene and $F_{off}$ is the feature code in the feature code database established offline.

**Figure 9:**
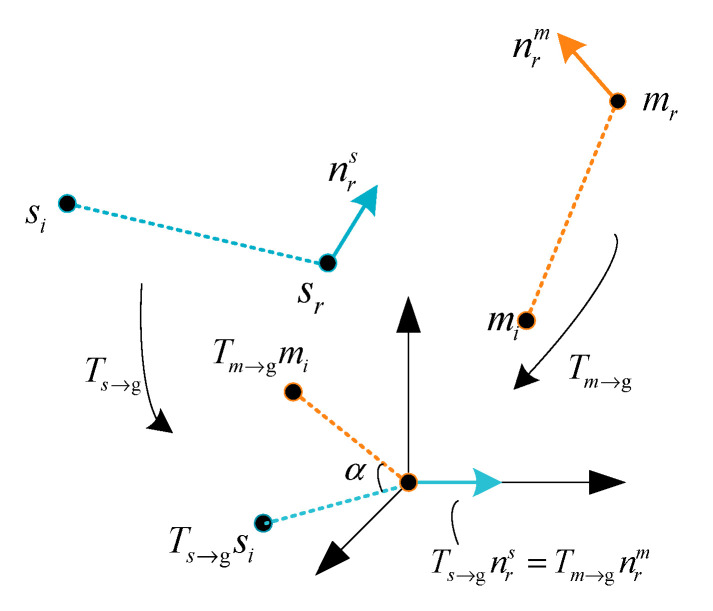
Model and scene coordinate system transformation.

We used a local coordinate system to vote in a two-dimensional space to determine the pose here proposed by Drost et al. [7]. We selected a reference point in the scene point cloud to form a point pair with any other point in the scene then calculated the feature value according to Formula (6) and searched for the point pair $\left(m_{r},m_{i}\right)$ in the feature code database through Formula (7). Successful matching indicates that feature point $s_{r}$ is extracted in the scene, where there is a point $m_{r}$ corresponding to it in the feature code database. We put them in the same coordinate system, as shown in Figure 9. Next, we moved these two points to the origin of coordinates and rotated them so that their normal vectors were aligned with the x-axis. Among them, the transformation matrix that occurs on $m_{r}$ is $T_{m\to g}$, the transformation matrix that occurs on $s_{r}$ is $T_{s\to g}$, and, at this time, the other points of their point pairs $s_{i}$ and $s_{r}$ are not aligned, and these need to rotate the angle to achieve alignment, with the transformation matrix is $R_{x}\left(\alpha \right)$, which then becomes the following [7]:

(8) $$s_{i}=T_{s\to g}^{-1}R_{x}(\alpha )T_{m\to g}m_{i}$$

where $T_{s\to g}^{-1}R_{x}\left(\alpha \right)T_{m\to g}\;$ is a temporary plank pose matrix.

In order to reduce the calculation time and increase the calculation speed, the rotation angle can be calculated by the following formula:

(9) $$\alpha =\alpha_{m}-\alpha_{s}$$

where $\alpha_{m}$ is only determined by the model feature point pair and $\alpha_{s}$ is only determined by the scene point pair features.

From Equation (8), it can be seen that the complex pose solving problem is transformed into the problem of matching model point pairs and corresponding angles α, so it can be solved by ergodic voting. We created a two-dimensional accumulator, where the number of rows is the number of scene model points M, and the number of columns is the value q after the angle is discretized. When the point pair extracted in the scene matches the point pair of the model correctly, one of the two-dimensional accumulators $\left(m_{r},\alpha \right)$ corresponds to it, that is, the position is voted.When all the point pairs composed of the scene point $s_{r}$ and other points $s_{i}$ in the scene have been processed, the position where the peak vote is obtained in the two-dimensional accumulator is the desired position. An angle α can estimate the posture of the plank, and the position of the model point can estimate the position of the plank.

**Figure 10:**
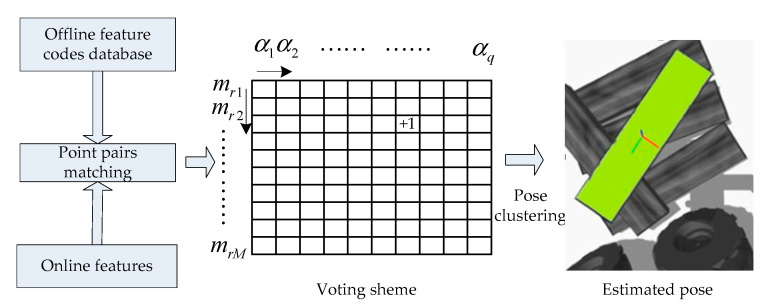
The final pose determination process of the plank.

In order to ensure the accuracy and precision of the pose, multiple non-repetitive reference points in the scene are selected to repeat the above voting. There are also multiple model points in the two-dimensional accumulator used for voting. In this way, there will be multiple voting peaks for different model points, eliminating significantly less incorrect pose votes, which can improve the accuracy of the final result. Multiple voting peaks means that the generated poses need to be clustered. The highest vote is used as the pose clustering center value. The newly added poses must have the translation and rotation angles corresponding to the pose clustering center pose values set in advance. Within a certain threshold range, when the pose is significantly different from the current pose cluster center, a new cluster is created. The score of a cluster is the sum of the scores of the poses contained in the cluster, and the score of each pose is the sum of the votes obtained in the voting scheme. After the cluster with the largest score is determined, the average value of each pose in the cluster is used as the final pose of the plank to be grasped. Pose clustering improves the stability of the algorithm by excluding other poses with lower scores, and at the same time ensures that only one pose is finalized during each recognition, so that the robot only chooses to grasp one wooden plank at one time. The method of obtaining the maximum score clustering pose average also directly improves the accuracy of the plank’s pose. This pose value can be used as the initial value of the iterative closest point method (ICP) [42] to further optimize the plank’s pose. In summary, the process to determine the pose of the plank is shown in Figure 10.

## 5. Experiments Results and Discussion

**Figure 11:**
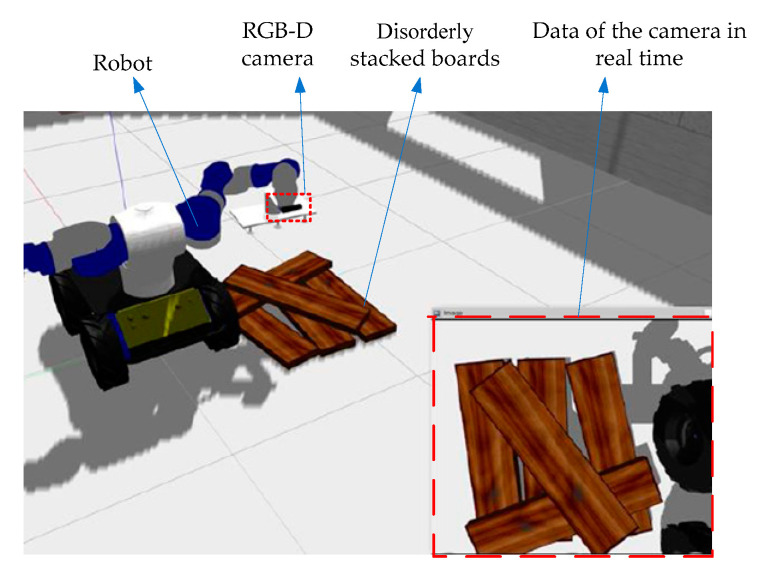
Visual grasping scene model of stacked wooden planks.

The computer hardware conditions used in the experiment were an Intel Xeon W-2155 3.30 GHz CPU, 16.00 GB of RAM, and a NVIDIA GeForce GTX 1080 Ti GPU. The whole framework is based on C++, OpenCV, Point Cloud Library (PCL) and other open source algorithm libraries. A visual grasp scene model of stacked wooden planks was built on the ROS (robot operating system) with the Gazebo platform. As shown in Figure 11, the RGB-D camera (Xtion Pro Live, Suzhou, China) is fixed at the end of the robot arm and the disorderly stacked wooden boards to be grasped are placed below. Besides that, the robot hand device is a suction gripper with six suction points.

### 5.1. Data Preparation and Convolutional Autoencoder Training

**Figure 12:**
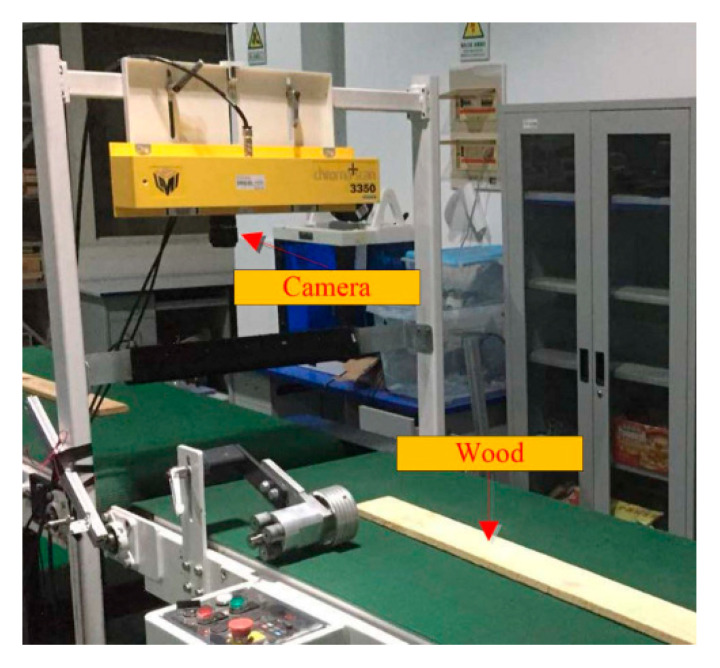
Wood image acquisition equipment.

**Figure 13:**
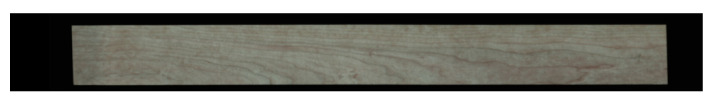
Collected image of the surface of a wood.

**Figure 14:**
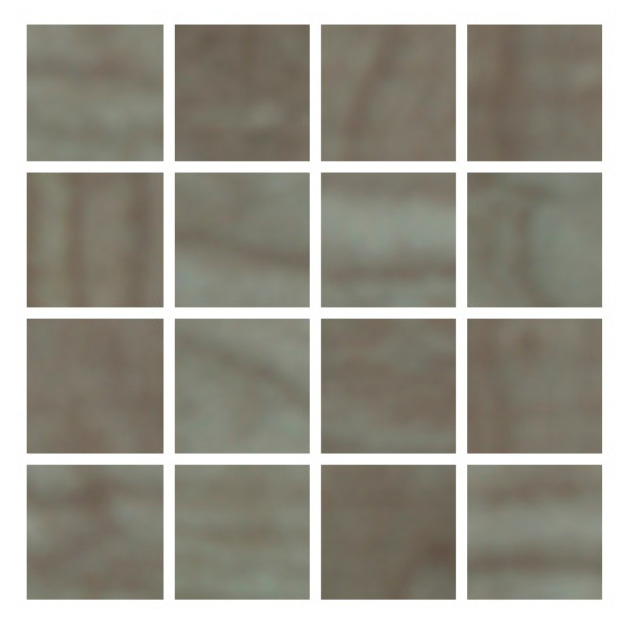
Some local images, which were intercepted from collected wood images.

For setting up the data set, we used the self-developed mechatronics equipment to collect the images of wood boards (Figure 12). This device mainly includes a strip light source, a transmission device, a CCD industrial camera (LA-GC-02K05B, DALSA, Waterloo, ON, Canada) and an photoelectric sensor (ES12-D15NK, LanHon, Shanghai, China) mounted on top. When the conveyor belt moves the wood board to the scanning position, the photoelectric sensor will detect the wooden board and start the CCD camera to collect the image of the wood board surface. We collected 100 images of red pine and camphor pine planks (Figure 13) and eventually divide them into small pieces of about 8000 local images (Figure 14).

The data collected by the self-developed equipment accounts for 75% of the whole data set, while the remaining 25% of the data set is collected in the ROS system. The different poses of the end of the robotic arm bring the RGB-D camera to collect scene information from different perspectives to obtain images of the same scene in different perspectives. First, the camera collects feature points at different positions from each image and intercept 22 × 22 pixels local image patches around the points. From the positive kinematics of the robot and the hand-eye calibration relationship, the pose of the camera can be known. When the scene is fixed, the corresponding points on the image under different perspectives can be known, and the local image patches are intercepted around the same corresponding point on the image under different perspectives. We took a set of two of them as the input end sample and output end expectation value of the training convolutional autoencoder and collected a total of 4000 sets of such local image patches.

**Figure 15:**
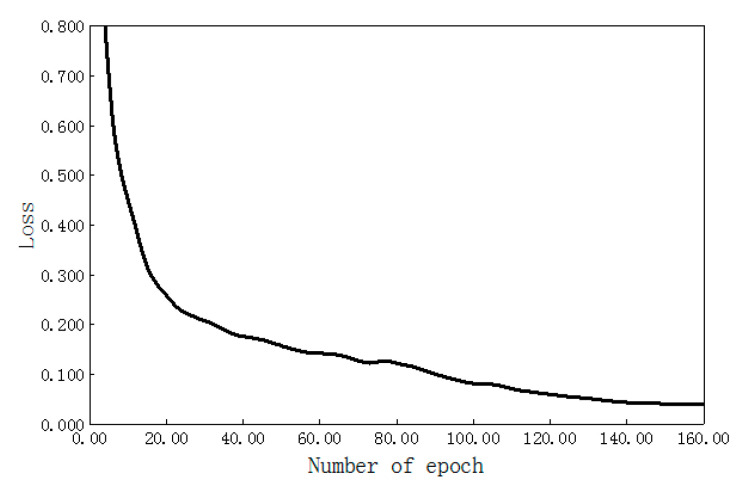
Iterative loss function curve of the deep convolution auto-encoder.

**Figure 16:**
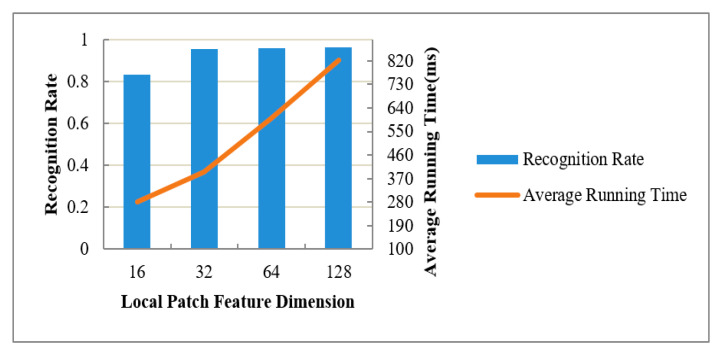
The influence of feature dimensions of the local image patch on recognition performance.

The network training used the deep learning autoencoder model designed in this paper. The structure size of each layer is shown in Table 1. The encoding stage contains four convolutional layers, and the decoding stage contains four transposed convolutional layers. The neural network training adopted the form of full batch learning, where the epoch is 160, and the relationship change curve between the training error and iteration number is shown in Figure 15. When the number of epochs was less than 20, the loss value of the network model decreased faster. When the number of epochs was more than 20, the loss value of the network model decreased slowly. When the epoch was 120–160, the loss value of the network model remained basically stable, that is, the model converges. Through training the network model, a 32-dimensional local texture feature descriptor of the wood plank that was stable enough for viewing angle changes was finally obtained. During the experiment, four feature dimensions of local image patches (i.e., 16, 32, 64, and 128) were specifically tested, and recognized 600 planks with different poses. We used the final pose to meet the pose accuracy of the plank to be grasped as the correct pose for recognition. We calculated the recognition rate and pointpair matching time to evaluate the performance of these four feature dimensions, as shown in Figure 16. With the 16-dimensional, 32-dimensional, 64-dimensional, and 128-dimensional feature dimensions increasing sequentially, in other word, the feature expression code was more abundant, so the recognition rate of the wood plank gradually increased, i.e., increases of 83.2%, 95.6%, 95.9%, and 96.4%. After the feature dimension size reached 32 dimensions, the recognition rate was not significantly improved. An increase in feature dimension size was also accompanied by an increase in computing time. When the feature dimensions were 64 dimensions and 128 dimensions, the calculation time of the plank pose increased even more. Considering the recognition rate and computing time, the 32-dimensional local image feature descriptor of the wooden plank was finally selected.

### 5.2. Grasping of Planks

**Figure 17:**
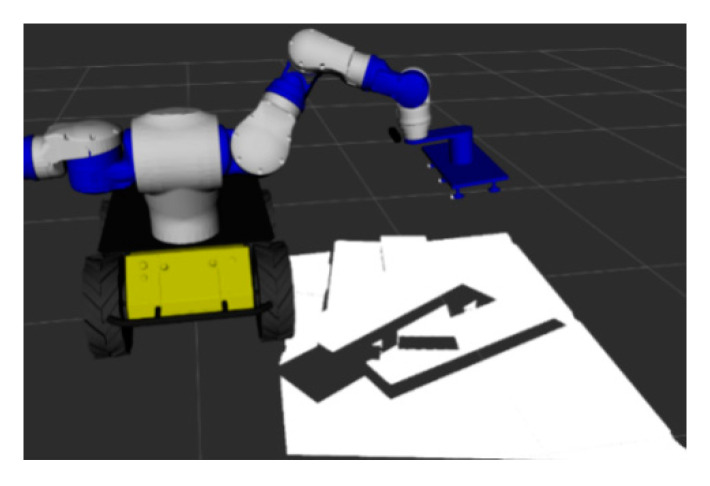
Original point cloud.

**Figure 18:**
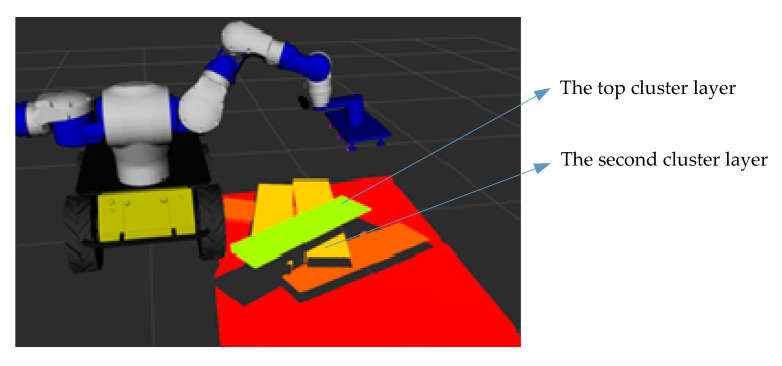
The segmentation result of point cloud after regional clustering.

The robot first grasps the top plank of the stacked plank. The original point cloud visualization result of the stacked plank scene under the RGB-D camera using the Rviz tool is shown in Figure 17. Combining the Euclidean distance clustering method [41], the point cloud under the camera was divided into different areas, so that different planks correspond to different point cloud clusters. The visualization result using the Rviz tool is shown in Figure 18. The cluster with the smallest average depth value was confirmed as the current priority grasping area, and the point pair matching was completed using the aforementioned method based on pointpair features and local image patch features. Then, we performed pose voting and clustering and finally determined the pose ${}_{o}^{c}M$ of the plank to be grasped.

After obtaining the pose ${}_{o}^{c}M$ of the plank to be grasped in the camera coordinate system, the current pose ${}_{h}^{b}M$ of the robot end (by forward kinematic solution of the robot kinematics) and the result ${}_{h}^{c}M$ of the robot hand-eye calibration, the plank’s pose was converted to the robot base coordinate system:

(10) $${}_{o}^{b}M={}_{h}^{b}M{{}_{h}^{c}M}^{-1}{}_{o}^{c}M$$

**Figure 19:**
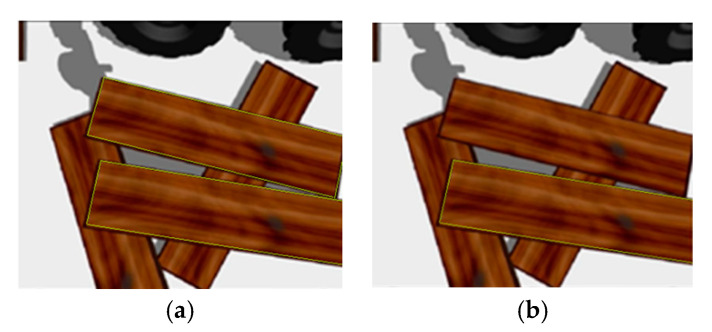
Wood plank recognition results: (**a**) Recognition results of some wood planks in the same cluster; (**b**) The board to be grasped.

**Figure 20:**
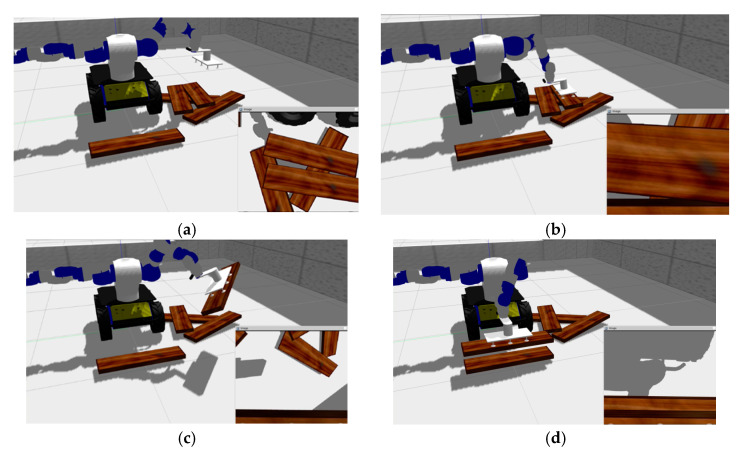
The result of the robot visually grasping wooden planks: (**a**) Identifying the pose of the plank to be grasped; (**b**) positioning the end of the robot and grasping; (**c**) during the robot handling process; (**d**) preparing to place the grasped wooden plank.

Then the grasping operation was realized by driving the end of the robotic arm to move to this position. As shown in the Figure 18, the top cluster has only one piece of wood, which is easy to locate and grasp with our method. When the robot hand has grasped and moved away the top board, the original second cluster layer is now the top position. The cluster contains two planks, since the two planks are close together. As shown in Figure 19, the two planks are not fully presented in camera vision and their respective image dose not include the whole plank, which is similar to the occlusion effect. In this case, our method can also obtain the recognition results of the cluster with multiple boards and select the target to be grasped (Figure 19). The multiple pose voting peak obtained by PPF algorithm get the poses of multiple wood planks in the same cluster and the pose with the highest voting score is selected as the target pose to be grasped by robot. As shown in Figure 20, the recognition and positioning of the plank to be grasped is accurate, and the robot grasping action process is smooth.

We carried out a grasping experiment 1000 times on randomly placed wooden planks in stacked wooden planks piles, also using other methods to perform the same number of experiments, and then compared the recognition rate, average recognition time, and grasping success rate. If the positioning accuracy of the recognition result is less than 3 mm and the rotation angle error is less than 2°, this situation is good for grasping success. This positioning accuracy is regarded as the correct recognition. As is shown in Table 2, the corresponding recognition performance comparison is shown in Figure 16, where “PPF” [7] was the traditional point pair method; “CPPF” [14] used the pint pair feature added color information. “SSD-6D” [17] used the convolutional neural networks for end-to-end training to obtain the pose of an object. “LPI” only used the local image patch proposed here to match the feature points and ICP calculated the pose, and did not use the point pair method to match; “LPPPF” is a method we proposed to determine the pose based on the local image patch combining point pair feature matching feature points, pose voting, and clustering.

**Figure 21:**
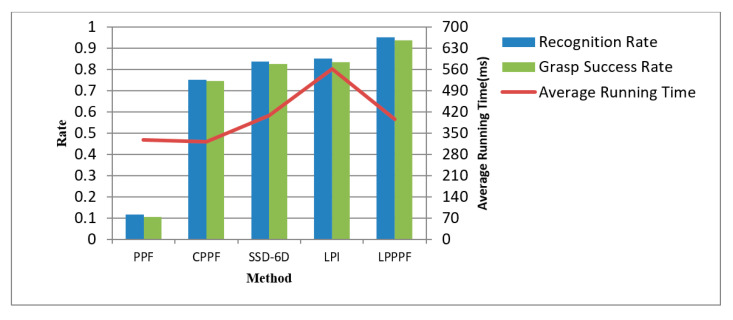
Comparison of the recognition performance of different methods.

From Table 2 and Figure 21, it can be seen that the recognition rate of the plank is closely related to the success rate of robot grasping. The higher the recognition rate, the greater the success rate of robot grasping. The feature descriptors that only use PPF methods feature poor description of the surface features of the wood plank, so the recognition rate is significantly lower. Even if color information is added to the traditional point pair feature in CPPF method, color information is only one of the features of the plan and the recognition rate of this method is not high here. SSD-6D using convolutional neural networks for end-to-end training to obtain the pose of an object also does not have a high recognition rate because of the low positioning accuracy. The local image patches used only have certain advantages in describing wood texture features, and the recognition rate has been improved to a certain extent. However, the feature description is not comprehensive enough, resulting in a low recognition rate 85.1%. The LPPPF method we proposed here has a certain improvement in the recognition rate of the wood plank to be grasped compared to other methods, which is about 11 percentage points higher when using deep learning SSD-6D method. Compared with only using local image patch features, it is about 9 percentage points higher. Additionally, the average computing time is also relatively short, i.e., 396 ms. This shows that this method has obvious application advantages in grasping occasions in the scene of disorderly stacking planks. Through the convolutional autoencoder to extract the texture features of the local image patches of the wood, combined with the point pair features, the surface features of the wood can be better expressed. At the same time, in view of the hierarchical nature of the wood stacking, an Euclidean distance clustering method is used for segmentation first, which avoids the entire scene for collecting image patches for matching, greatly reducing the number of local image patches that need to be extracted and ultimately reducing the calculation time for recognition.

## 6. Conclusions

The main shape of a plank is a large plane that is symmetrical and regular. The current conventional methods make it difficult to identify and locate planks to be grasped in scenes of disorderly stacked planks, which makes it difficult for robots to grasp them. A recognition and positioning method combining local image patches and point pair features was proposed here. Image patches were collected from disorderly stacked wooden boards in the robot vision scene and a convolutional autoencoder was used for training to obtain a 32-dimensional local texture feature descriptor that is robust to viewing angle changes. The local image patches around the point pair from the single-plank model were extracted, the feature code was extracted through the trained encoder, and the point pair geometric features were combined to form a feature code describing the feature of the board. In the stacking plank scene, the area of the plank to be grasped was segmented by a Euclidean distance clustering method and the feature code was extracted, and the plank to be grasped was identified through processes such as matching point pairs, pose voting and clustering. The robot grasping experiment here has proven that the recognition rate of this method is 95.3%, and the grasping success rate is 93.8%. Compared with PPF and other methods, the method presented here has obvious advantages. It is suitable for the grasping of disorderly stacked wood planks. At the same time, it has certain reference significance for recognition and grasping in other similar conditions.

## Figures and Tables

**Table 1:** Deep convolutional auto-encoder construction.

	Connection Layer	Filter Size	Feature Size	Stride Size	Activation Function
Coding stage	Input layer	——	22 × 22	——	——
	Convolution layer 1	3 × 3	20 × 20 × 128	1	Relu
	Convolution layer 2	3 × 3× 128	18 × 18 × 128	1	Relu
	Convolution layer 3	3 × 3 × 128	9 × 9 × 256	2	Relu
	Convolution layer 4	9 × 9 × 256	1 × 1 × 32	——	Relu
Decoding stage	Transposed convolution 1	9 × 9 × 32	9 × 9 × 256	——	Relu
	Transposed convolution 2	3 × 3 × 256	18 × 18 × 128	2	Relu
	Transposed convolution 3	3 × 3 × 128	20 × 20 × 128	1	Relu
	Transposed convolution 4	3 × 3 × 128	22 × 22	1	Relu

**Table 2:** Performance of different combination methods in grasping wood planks. PPF: Point pair feature.

	PPF	CPPF	SSD-6D	LPI	LPPPF (Proposed Method)
Recognition rate	0.115	0.753	0.837	0.851	0.953
Average operation time (ms)	327	322	409	563	396
Grasping success rate	0.105	0.746	0.825	0.835	0.938
